# Toward Coordinated Colloids: Site-Selective Growth of Titania on Patchy Silica Particles

**DOI:** 10.1038/srep09339

**Published:** 2015-03-23

**Authors:** Changdeuck Bae, Hyunchul Kim, Josep M. Montero Moreno, Gi-Ra Yi, Hyunjung Shin

**Affiliations:** 1 Department of Energy Science, Sungkyunkwan University, Suwon 440-746, South Korea; 2 Integrated Energy Center for Fostering Global Creative Researcher (BK 21 plus), Sungkyunkwan University, Suwon 440-746, South Korea; 3 Institute of Applied Physics, University of Hamburg, Jungiusstrasse 11, Hamburg 20355, Germany; 4 Department of Chemical Engineering, Sungkyunkwan University, Suwon 440-746, South Korea

## Abstract

Rational synthesis of coordinated spherical colloids is reported by site-selective growth of secondary hemispherical patches on primary spherical particles with quasi-defined coordination numbers and positions. We clarify the importance of mass transport phenomena on the site-specific secondary nucleation/growth in nanoparticulate colloidal systems. By comparing ultrasonic and conventional agitation during patch growth, we found that enhanced mass transfer is the key to controlled, homogeneous transport of the molecular precursors in a solvent onto the nanoparticles. With chemically defined nucleation sites, the surfaces of spherical silica particles were modified for use as a new kind of colloid with patches at desired coordination positions. Our observations represent a significant breakthrough in colloidal chemistry and self-assembly.

More complex molecular and colloidal building blocks can spontaneously organize into ordered arrangements via self-assembly to form previously unknown meso-scopic lattice structures^1^,^2^,^3^,^4^,^5^,^6^,^7^. Such studies are of significant importance not only for the understanding of defect dynamics and phase transitions of atomic lattices^8^,^9^,^10^,^11^,^12^,^13^,^14^, but also in advanced optics such as plasmonics^15^,^16^,^17^ and photonics^18^ or for a new generation of catalysis^19^,^20^. Over the past decade, synthesizing colloidal particles has been greatly successful with many unconventional shapes^21^,^22^,^23^,^24^,^25^,^26^,^27^,^28^,^29^,^30^,^31^,^32^,^33^,^34^,^35^,^36^,^37^,^38^,^39^,^40^,^41^,^42^,^43^,^44^,^45^,^46^. In order to achieve more complex, mesoscopic crystals, in contrast, the self-assembly of these particles is in its infancy. Therefore, colloids with functional patches at designated positions are highly desired. The spectrum of applications includes a large variety of possibilities, i.e. the chemical industry, highly efficient, customized solar cells and fuel cell devices, and optical and optoelectronic devices.

One approach was to aggregate mono-dispersed, spherical polymeric particles into several clusters, for example, using oil-in-water emulsion methods. Upon size selection processes, colloidal clusters with diverse geometries can be prepared^47^,^48^. The other strategy was to pattern the surfaces of colloids by physical vapor deposition (at glancing angles), producing so-called Janus and patchy particles^49^,^50^,^51^.

Previously, we proposed a general concept for producing so-called coordinated colloids (CCs), and demonstrated it by fabricating titania@silica CCs^52^. Briefly, mono-dispersed Stöber silica particles were crystallized on glass substrates, followed by coating with octadecyltrichlorosilane (OTS) self-assembled monolayers (SAMs). Then, the topmost layers were delaminated, and the remaining patterned monolayer was ready for secondary patch growth. The titania patches were thus only grown on the opened OTS-SAM patterns, working as nucleation sites via a sol-gel route. For the resulting titania@silica CCs, the number of patches was three or four, in accordance with the orientation of the facets of the colloidal crystals (see, Fig. 1C). Note that the silica colloidal crystal films were immobilized on the glass substrate during the secondary reaction, as schematically depicted in Fig. 1A.

## Results and Discussion

**Figure 1:**
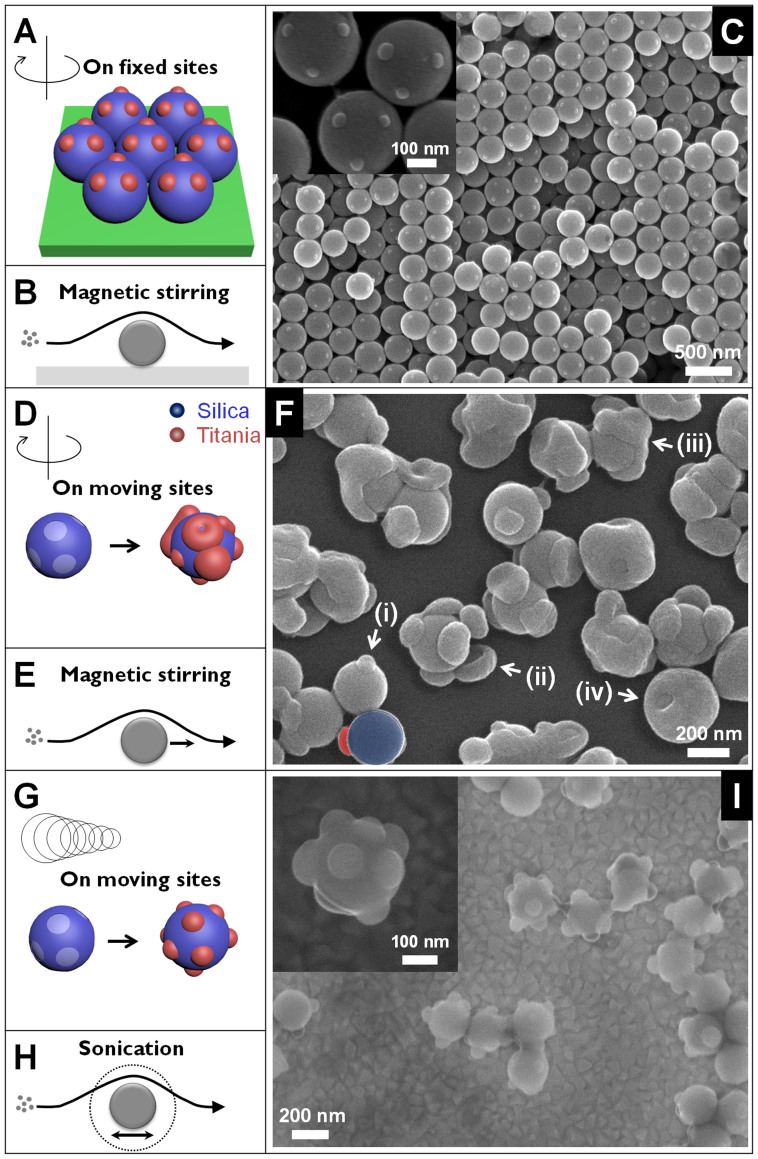
Comparison of three different kinetic conditions used in the synthesis of coordinated colloids (CCs). (A–C) Primary spherical particles of silica (marked as blue) with OTS-SAM openings (marked as pale blue) on their surfaces are fixed on the underlying substrate and subjected to the growth of secondary patch (marked as red) under magnetic stirring (Ref. 52). (C) Representative SEM images of the resulting CCs. (D–F) Primary particles with OTS-SAM patterns are suspended in a solvent, and the identical experimental conditions with those in panel A–C are applied, producing CCs. (F) The corresponding SEM micrographs show secondary titania patches at different levels of maturity (i through iv). (G–I) Experiments as in panel D–F, except under ultrasound irradiation, and (I) the resulting CCs where the secondary titania patches were grown on the primary silica spheres with high fidelity at given coordination positions (inset, magnified view). (B, E, and H) Schematic depictions in grayscale for the visualization of mass transports in each case (i.e., B: fixed nucleation sites + magnetic stirring, E: moving nucleation sites + magnetic stirring, H: moving nucleation sites + sonication), where small spheres represent precursor molecules and bigger ones are primary particles for nucleation.

The question naturally arises whether our idea could be generalized to individual colloids suspended in a solvent. We have applied this process to well-separated silica particle suspensions with the same defined nucleation sites in OTS-SAM under identical growth conditions (i.e., agitation by magnetic stirring), as shown in Fig. 1D. The detailed sol-gel experiments can be found in our previous paper^52^. Remarkably, we were not able to grow the secondary patches uniformly, in contrast to a previous report by others^53^. Fig. 1F shows the result of titania@silica CCs. The secondary patches are found at different stages of maturity, marked by (i) through (iv) in Fig. 1F. They also differ from the primary silica beads with OTS-SAM patterns on them in terms of not only the uniform morphology (Fig. S1 in the supporting information, SI), but also the elemental analysis (Fig. S2 in SI).

In the case of immobilized particles, the transport originated by the stirring of the solvent assures a sufficient supply of precursors to the nanoparticle during TiO_2_ patch growth. However, the suspended particles move collectively, pulled by the stirred solvent, so that the relative flow between the solvent and the particles is in comparison reduced (also marked as black arrows in Figs. 1B, E, and H). In such a situation, the above-mentioned observation is consistent with Einstein's analysis of Brownian motion, where bigger particles in fast-moving molecules of a solvent are assumed to follow random walks^54^,^55^,^56^. This is on the basis of an extreme separation of timescales between the two. However, the scenario might fail if the size of the bigger particles is small down to a certain diameter, as in our systems. The timescales of the two Brownian motions are comparable between each other, resulting in their collective movements during growth. That is, the precursor molecules could not be homogeneously diffused to the moving, bigger particles (here, nanoobjects as in Fig. 1E), and the concentration gradients were not at the steady state. The resulting patches were not only different in size (for example, Fig. 1F i and ii), but also grew at broken equilibrium in the surface tension at three phase contact lines, as indicated in Fig. 1F (iii and iv).

**Figure 2:**
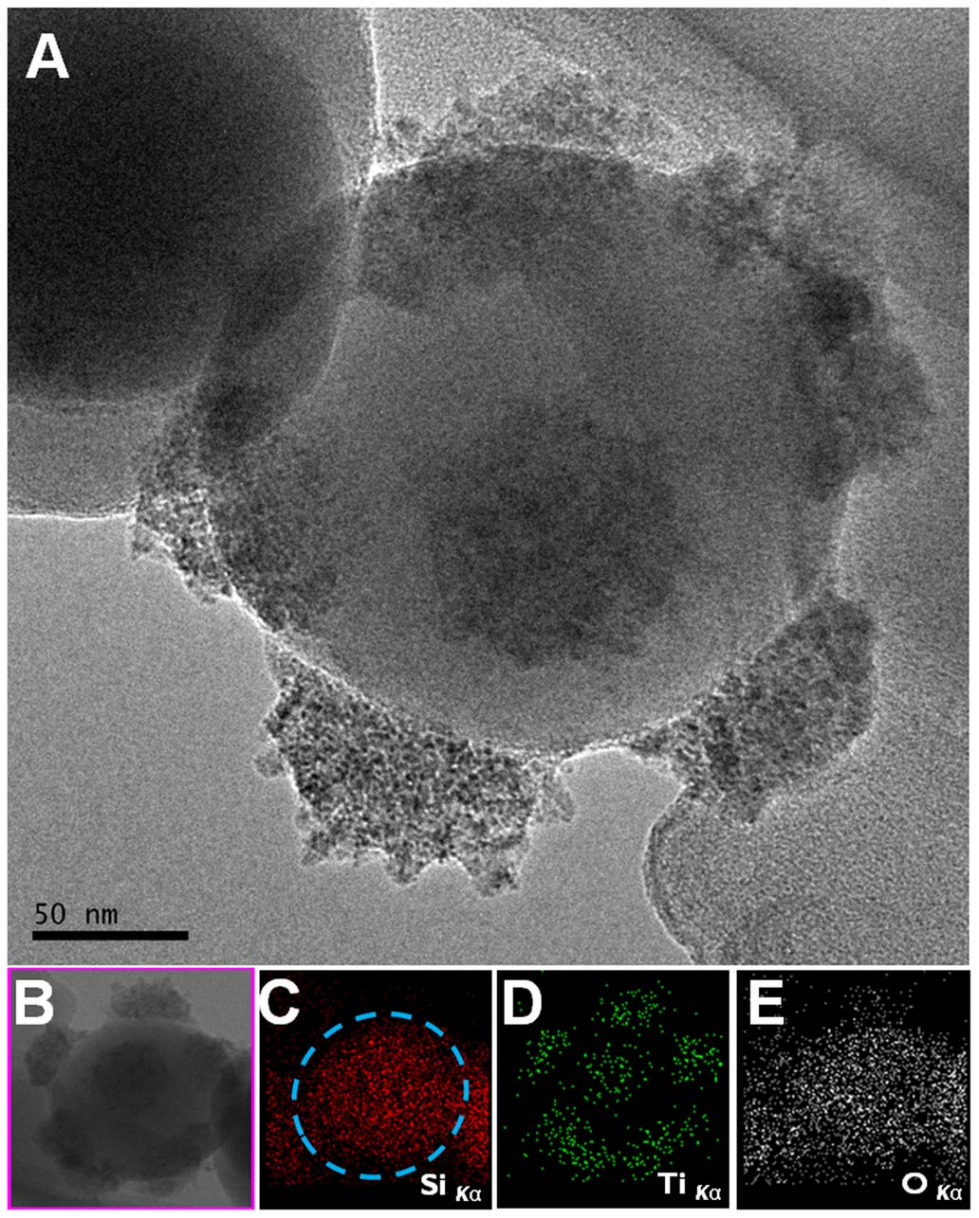
(A–E) TEM analysis of a representative titania@silica CC upon thermal treatments: (A) Bright-field TEM; (B) Scanning TEM; (C–E) Elemental maps.

**Figure 3:**
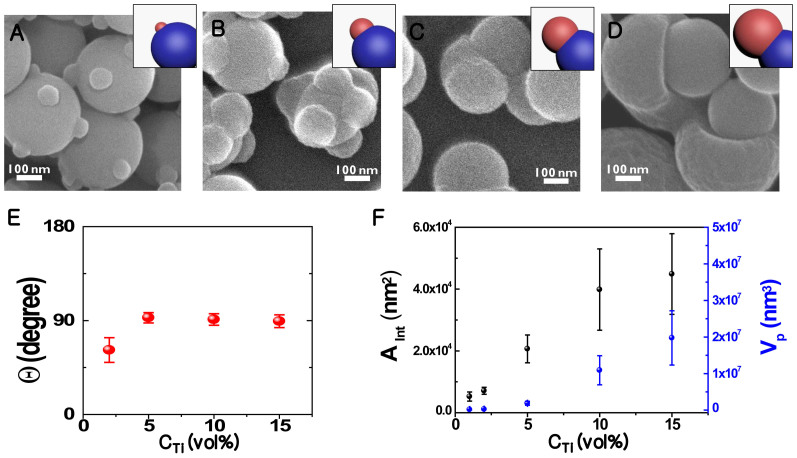
Evolutionary patch growth with increasing precursor concentrations C_Ti_ (vol%). (A–D) The representative SEM micrographs at different C_Ti_. (E) The equilibrium contact angle ***Θ***, and (F) the measured interface area ***A***_int_ and volume ***V***_p_ of the secondary patches on the silica body.

Next, we show how the issues of non-uniformity and non-reproducibility, essential in this line of research, can be overcome by the enhanced mobility of bigger objects under an ultrasonic wave field (40 kHz, 160 W) during secondary growth for ~1 hr (Fig. 1G). Fig. 1I shows the result of titania@silica CCs. Compared to that by magnetic stirring (Fig. 1F), uniform-sized growth of the secondary titania patches was achieved with high fidelity at given coordinated positions. Moreover, elemental mapping revealed a clear distribution of secondary patches (Fig. 2). The particles were analyzed upon thermal treatment at 400°C for ~1 h, as amorphous titania could have been damaged by the electron beams in TEM. It is worth mentioning that the fidelity in the number of patches rather pertains to the initial quality of silica opal structures. Silica particles deposited by capillary self-assembly typically have a face-centered cubic (fcc) lattice structure. This is originated from a combined effect of the spherical shape and the free energy minimization during growth. As such, each particle is expected to have 12-fold coordination. However, imperfect connections between spherical building blocks at the coordinated positions were often observed in the inverse opals produced from fcc opals without OTS-SAM. Equivalently, missing patches on CCs were found as well, shown in Figs. 1I and 3. Polydispersity and the subsequent displacements between silica beads inside the lattice are the major causes of missing patches, even though the polydispersity of our silica colloids (200 nm in mean diameter) was less than 3%. Indeed, 8–10 patches in average were found by measuring about 30 CCs at appropriate facing angles when analyzing electron micrographs, as shown in Fig. S4 (see SI). Note that the coordination numbers according to the corresponding secondary growth were manifested without slightly sintering them, in contrast to a previous report by others^53^.

The hydrodynamics in real systems is more complex in that the driving forces for mass transport phenomena involve convection and diffusion as well. For example, ultrasonication is likely to result in local heating, which may increase the diffusion coefficient of the molecular species. Even though individual contributions are not clear at the current stage, ultrasonic treatment seems to enhance mass transfer coefficients in all processes, improving monodispersity by facilitating the access of reactants and removing the reaction products. An additional point of view is that ultrasonication may open new reaction paths which are usually not available due to a high energy barrier; ultrasonication may help to overcome this by providing sufficient energy to the precursor molecules. Therefore, nucleation events likely take place more homogeneously on the surfaces in a spatial manner.

We suspect that the enhanced mobility of primary particles under an ultrasonic wave field recovered the mass transfer conditions for a sufficient distribution of the reactants on the surfaces of the particles. In sonochemistry, it is well-known that, during ultrasound irradiation, cavitation and shock waves can accelerate solid particles to high velocities, and the speed of particles in a colloidal suspension can reach up to several kilometers per hour^57^,^58^. An enhancement in mass transfer coefficients has been experimentally reported by Atobe et al.^59^ Such systems have been modeled by Luo et al.^60^, where the solvent is treated as a series of finite elements carrying the reactants to the particles by flowing along the boundary layer of the particle with a velocity given by two components: a constant speed and a frequency-dependent speed^60^. This simple model accounts for the enhancement in mass transfer coefficients, ***ξ*** when ultrasonication was used (also see SI). The model successfully predicts the influence of experimental parameters, for example, an increase of the sonication frequency results in a decrease in the mass transfer coefficients, while a higher power of the ultrasound significantly increases the efficiency.

In a simplified equation derived (see SI for more details), one can see the dependence ***ξ*** as a function of the frequency and the power of the ultrasonication. Since the ultrasound power is in direct proportion to ***V***_u_ (W ∝ *V*_u_^2^), from the equation, one can see that ***ξ*** increases with the power (***ξ*** ∝ W^1/4^). On the other hand, as ***k*** is in direct proportion to the sonication frequency (***k*** ∝ ω^1/2^), then ***ξ*** decreases with frequency. This is explained by particles being unable to follow the agitated flow at very high frequencies, such that the benefit of ultrasonication on the mass transfer coefficient is lost. Therefore, we think that the local equilibrium in the precursor concentration around the nucleation sites reached nearly a steady state thanks to the enhanced mass transfer coefficient. Indeed, such an explanation was evidenced by observing the robust site-selectivity while varying the initial concentration of the precursor, as follows.

Fig. 3 shows the evolutionary growth of patches of titania@silica CCs by varying the initial concentration of Ti-containing precursors, ***C***_Ti_ (defined as ***V***_TBT+EG_/***V***_Acetone+Water_ where TBT respectively denotes titanium *tert*-butoxide, and EG, ethylene glycol) in the patch growth. In contrast to the results with normal agitation (Fig. 1F), the secondary patches were grown in a site-specific manner up to very large patches with increasing ***C***_Ti_. Synchronized, homogeneous and uniform-sized growth of the patches is observed in all formation stages. The site-selectivity and the homogeneity might be quantitatively described by monitoring the physical dimensions of titania patches such as their contact angle ***Θ*** and contact area ***A***_int_ to the primary silica spheres (see also Fig. S3 in SI). In our case, the three phase contact lines were defined between the primary silica particle with OTS-SAMs (O), the secondary sol-gel titania hemisphere (G), and the precursor-solvent mixture (P). If the silica-solvent interfacial energy is denoted by ***γ***_OP_, the silica–titania interfacial energy by ***γ***_OG_, and the titania–solvent interfacial energy by ***γ***_GP_, the equilibrium contact angle ***Θ*** is determined by Young's Equation:
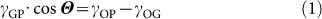
The surface tension equilibria could be experimentally determined by ***Θ***. It is worth noting here that low contact angles are expected typically in the oxides' growth on oxide surfaces like in our titania on silica, as shown Fig. S6 in SI. With the presence of OTS-SAMs, however, ***Θ*** was nearly kept to be high at the three phase contact lines (Fig. 3E) with varying ***C***_Ti_. Indeed, the observed ***Θ*** values were all close to 90° although the interface area ***A***_int_ and the resultant volume ***V***_p_ of the secondary patches were proportional to ***C***_Ti_ as expected (Fig. 3F). This implies that the site-selectivity operates effectively during the patch growth. The present strategy underscores the robust utility of OTS-SAM to control the nucleation and growth likely by modifying the surface energies around the nucleation sites when designing and growing coordinated colloids.

In summary, CCs have been conceptually demonstrated by some of the authors, previously that were inspired by atomic lattice structures^52^. However, generalizing our concept into fully coordinated patchiness has been limited by Brownian motion, in contrast to a previous report^53^. Here, we demonstrate a general method for creating CC particles, while avoiding size-selection processes^47^,^48^. The importance of Brownian motion on nanoparticular colloidal systems and their site-specific modification was revealed. Enhanced mobility of the bigger objects was found to be the key to controlling homogeneous diffusion of smaller molecular precursors in a solvent by comparing ultrasound radiation and conventional agitation during patch growth. Our results might indicate a new path in colloidal chemistry and self-assembly.

## Methods

### Fabrication of Primary Colloids

Silica particles were synthesized through the modified Stöber method^16^. Tetraethoxyl orthosilicate (TEOS, 99.999%, Aldrich), ethanol (anhydrous, Carlo Erba), and ammonia (28–30% solution, Junsei Chem., Japan) were commercially available and used without further purification. A mixture of 5 mL TEOS and 30 mL ethanol was injected into a mixture of 9.5 mL NH_4_OH and 60 mL ethanol under vigorous magnetic stirring at a constant feeding rate using a syringe pump. The average diameter (*D*_avg_) of the silica beads was controlled by a feeding rate of ~0.1 mL·min^−1^. After ~16 hr, a white precipitate was harvested by centrifugation, followed by repetitive washing with ethanol and distilled water. This was then dried in a vacuum at 100°C overnight. *D*_avg_ was ~200 and 350 nm (standard deviation ~3%).

To grow a colloidal crystal, silica particles were redispersed in ethanol in a glass vial. A glass microscope slide was vertically submerged in the silica solution and then dried in an environmental chamber (R.H. 74% and 45°C) for approximately ten days. OTS-SAMs were grown by immersing the samples in anhydrous toluene solutions containing 0.1 vol% OTS for several days under ambient conditions. OTS (95%, Aldrich) and toluene (99.8%, anhydrous, Aldrich) were commercially available and were used as received.

### Patch Growth

In a typical synthesis, a mixture of 0.2 mL titanium *tert*-butoxide (TBT, Aldrich) and 30 mL ethylene glycol (EG, Duksan, Korea) was magnetically stirred overnight and used as a precursor solution. Samples for all three cases (Fig. 1 in the main text) were immersed in acetone (anhydrous, Carlo Erba) containing a small amount of water (typically 0.1 mL). Desired amounts of the precursor solution (from 0.8 up to 6 mL) were immediately added to the acetone medium (see also Table S1). It is noteworthy to mention a possible that EG may react with the Ti alkoxide. That would reduce the reactivity of the original Ti alkoxide so that the possible intermediate would make the reaction of desire more controllable. At the current stage, unfortunately, we did not have any evidence. In this context, it is rational to define the precursor concentration C_Ti_ as the volume fraction (vol%) of C_Ti_ to the acetonic media, instead of the molar fraction of TBT in solvents. Gentle stirring (~300 revolutions per minute) and sonication (40 kHz, 160 W, Branson 1510, Emerson Electric Co. USA) as means of agitation were applied for a comparative study. After the desired reaction time, the samples were taken out of the solution and washed with pure ethanol and water several times.

### Characterization

The geometry and dimension of the resulting structures were investigated by field-emission scanning electron microscopy (FESEM, JSM7000F, JEOL, Japan) and high-resolution transmission electron microscopy (HRTEM, JEM2100F, JEOL, Japan). TEM samples were prepared by mechanically transferring the as-synthesized particles onto a substrate to a TEM grid. The map and spectroscopy by energy-dispersive x-ray spectrometry were recorded using an Oxford Inca Energy apparatus (Oxford Instruments, UK) equipped with JEM2100F.

## Author Contributions

C.B. and H.S. conceived the project. C.B. and H.K. carried out the synthesis and the characterization. C.B., H.K., J.M., G.Y. and H.S. analyzed data. J.M. derived the formula. C.B. wrote the paper. H.S. supervised the project.

## Supplementary Material

Supplementary Information SI
