# Supplementary material for: Toward Coordinated Colloids: Site-Selective Growth of Titania on Patchy Silica Particles
Source: Sci Rep. 2015 Mar 23;5:9339. doi: 10.1038/srep09339 (PMC4369688; doi:10.1038/srep09339)
Supplement: Supplementary Information — SI [file srep09339-s1.doc]

Supplementary Information

Toward Coordinated Colloids: Site-Selective Growth of Titania on Patchy Silica Particles

*Changdeuck Bae,^†,‡,*^, Hyunchul Kim,^†^ Josep M. Montero Moreno,^§^ Gi-Ra Yi^#^ and Hyunjung Shin^†,*^*

^†^ Department of Energy Science, Sungkyunkwan University, Suwon 440-746, South Korea

^‡^, Integrated Energy Center for Fostering Global Creative Researcher (BK 21 plus), Sungkyunkwan University, Suwon 440-746, South Korea

^§^ Institute of Applied Physics, University of Hamburg, Jungiusstrasse 11, Hamburg 20355, Germany

^#^ Department of Chemical Engineering, Sungkyunkwan University, Suwon 440-746, South Korea

* Corresponding Authors: changdeuck@skku.edu and [*hshin@skku.edu*](mailto:hshin@skku.edu)

**Table S1**. A summary of experimental conditions for preparing the samples at different kinetic conditions.

| Kinetic conditions | Methods | Primary Silica | Concentration of titania precursors (vol %)^†^ | The corresponding figures |
| --- | --- | --- | --- | --- |
| Case Ⅰ | Magnetic stirring | Fixed | 5 % | Fig. 1A-C |
| Case Ⅱ | Magnetic stirring | Moving | 5 % | Fig. 1D-F |
| Case Ⅲ | Sonication | Moving | 1 - 15 % | Fig. 3  (10 %, Fig. 1G-I) |

† A mixture of 0.2 mL titanium *tert*-butoxide and 30 mL ethylene glycol in acetonic solvent.


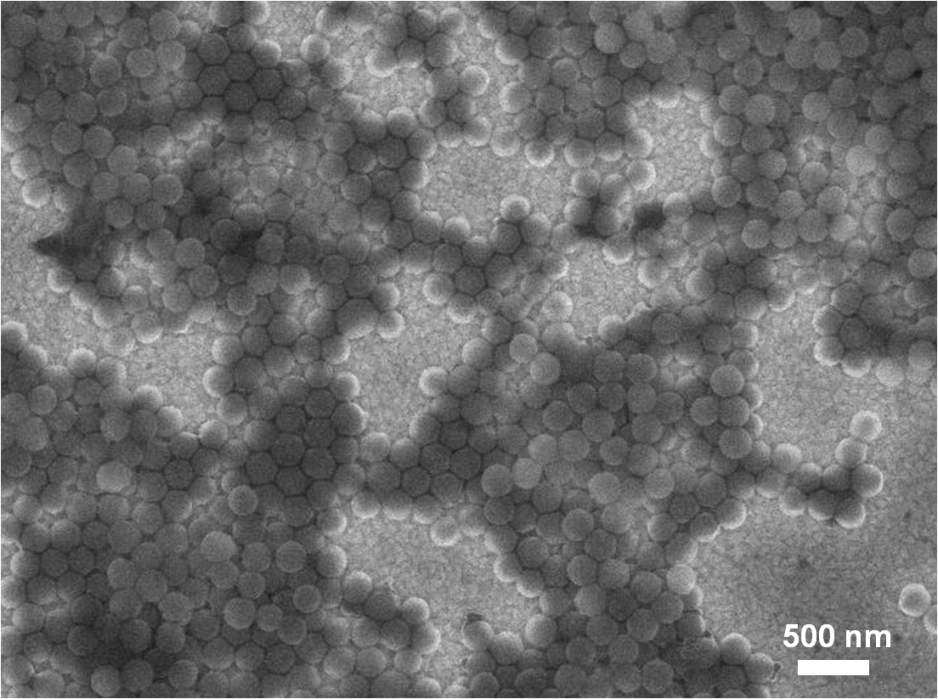


**Figure S1**. Representative SEM image of OTS-SAM-patterned Stöber silica particles. Uniform-sized particles before patch growth are seen on Pt substrate.


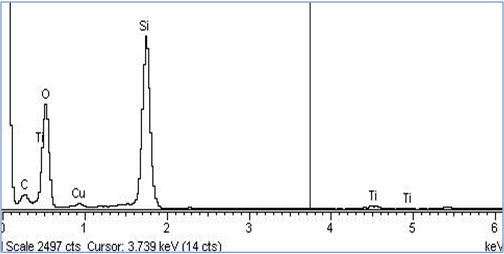


**Figure S2**. Typical energy-dispersive X-ray spectroscopy of titania@silica CCs.


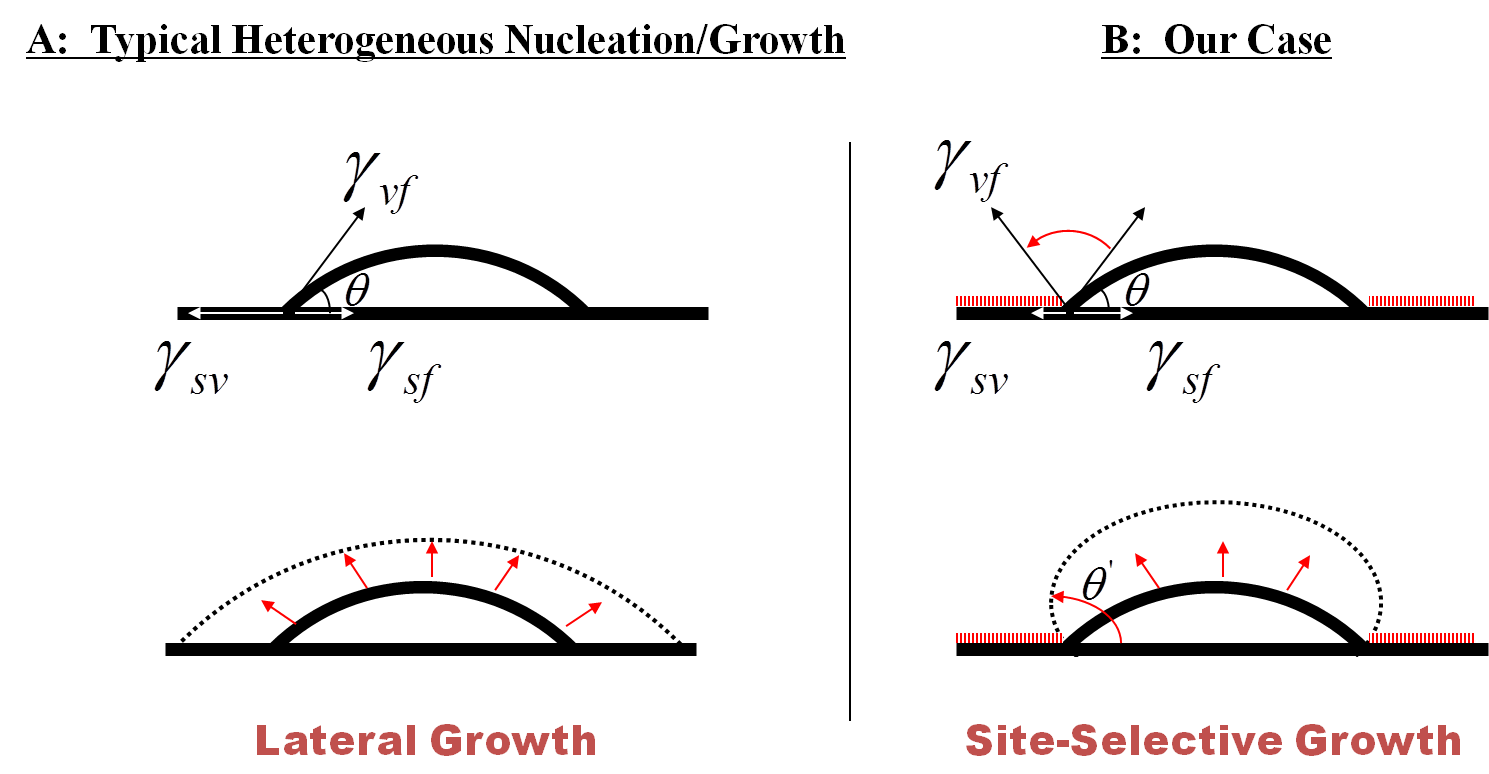


**Figure S3**. A comparison between (A) heterogeneous nucleation/growth and (B) site-selective growth at typical three-phase contact lines, where ***s*** stands for surface, ***v***, liquid, and ***f***, new phase. Rendering chemical contrasts such as our OTS-SAM patterns (marked as dotted red lines) should modify the surface energies (***γ***) as well as the contact angles (***θ*** → ***θ***'), directing/determining further growth as shown by the red arrows.


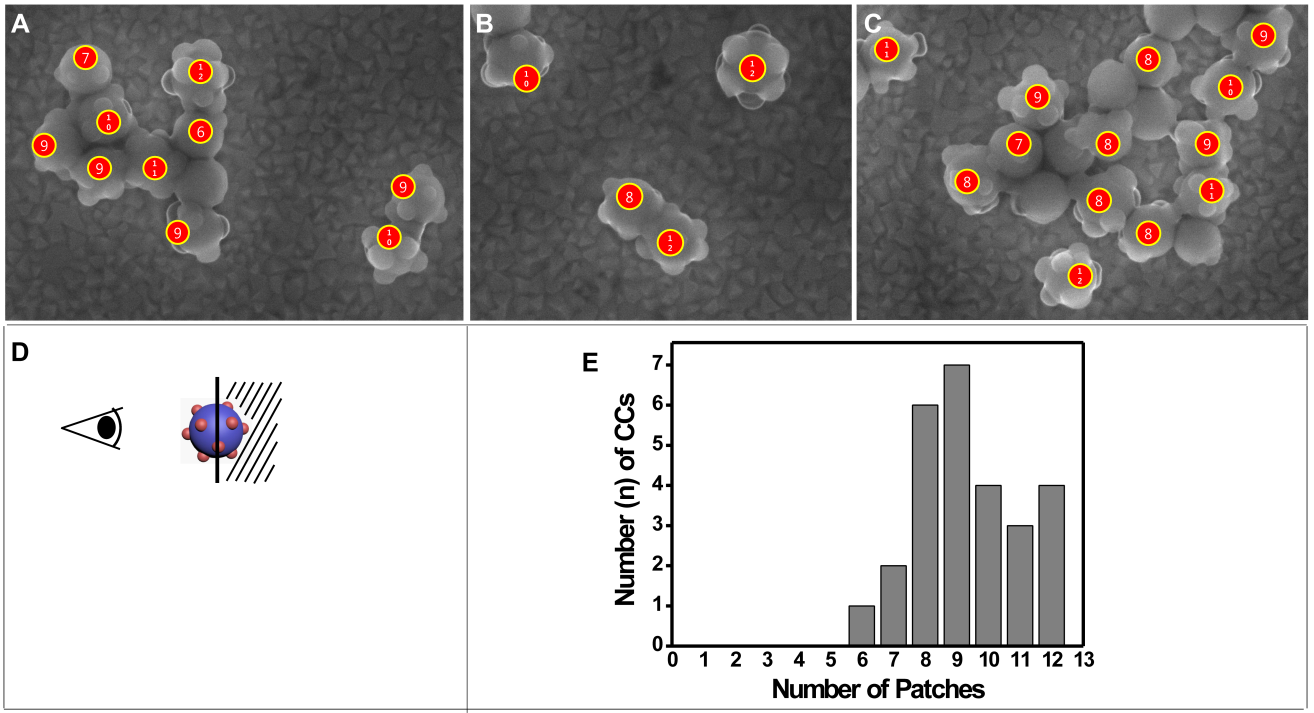


**Figure S4**. Statistical analysis on the number of patches in our titania@silica CCs. (A-C) Representative SEM micrographs of titania@silica CCs. (D) Consideration of unseen sides when counting the total patch number, and (E) the resulting histogram.


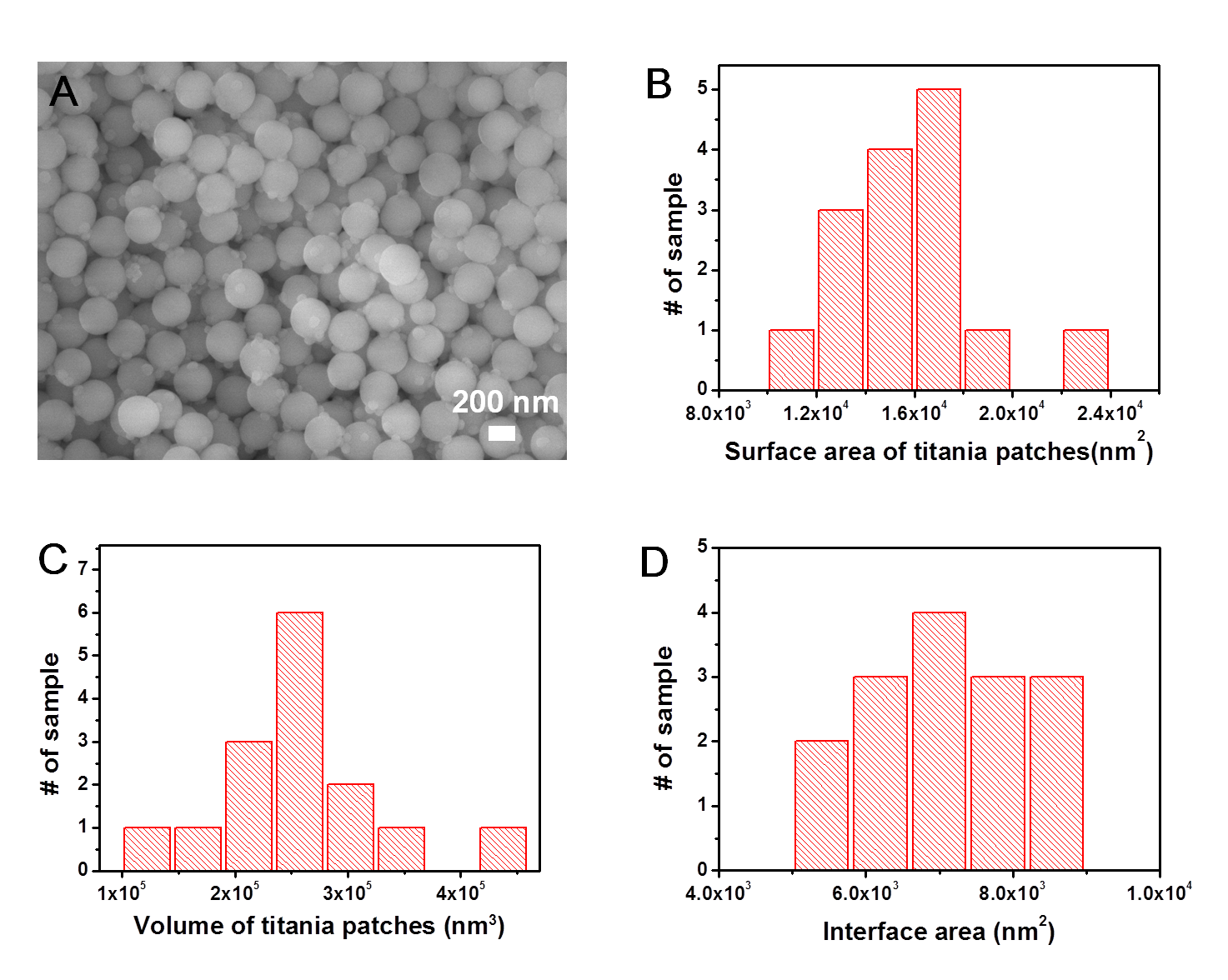


**Figure S5**. (A) Low-magnification SEM image of titania@silica CCs. (B-D) Related geometrical analysis between titania and silica parts, where detailed procedures are given below.


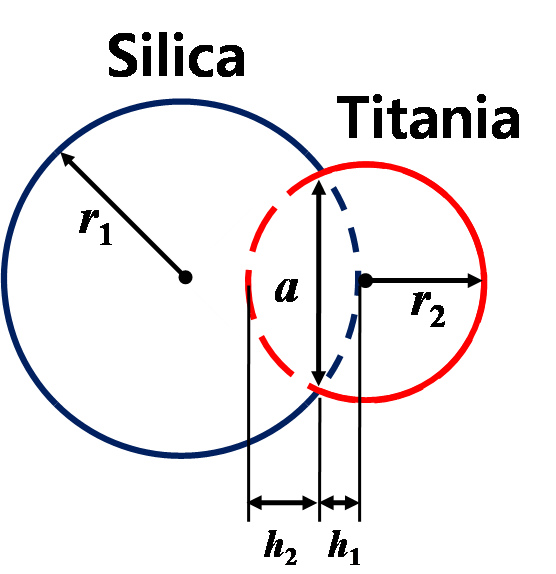


Volume of hemi-spherical titania is calculated by:

V_titania_= (Volume of titania-side sphere) – (Volume of overap)

=
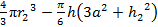


Surface area of interface is given by:


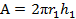


Surface area of the nanostructure

A _titania_= (surface area of sphere) – (surface area of overap)

=
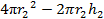


Contact angle is defined as follows:


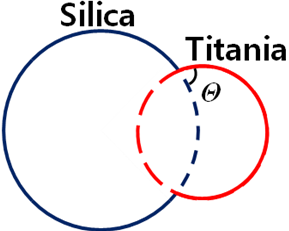


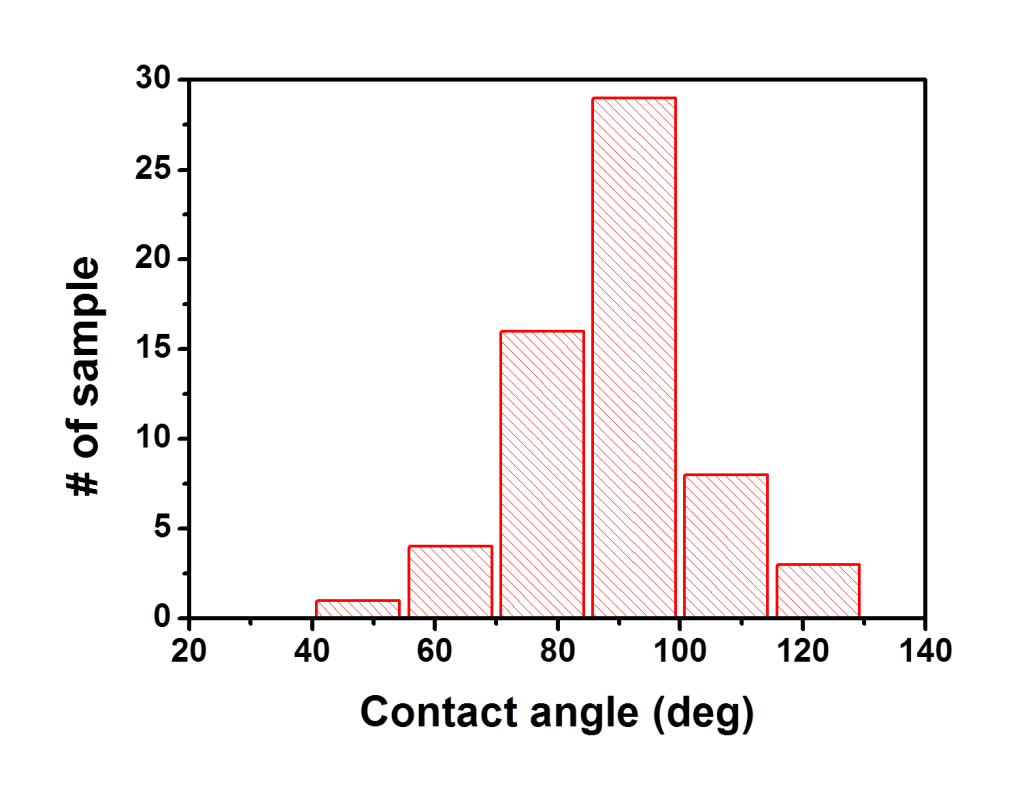


**Figure S6**. A histogram of contact angles, ***θ*** between titania and silica of CCs originating from a result of site-specific formation.

The above analysis is limited by the number of patches available because the patches oriented orthogonally to the viewing axis in SEM are possible to use in taking account for further geometrical estimation, and hidden and off-axis patches are discarded (see below).


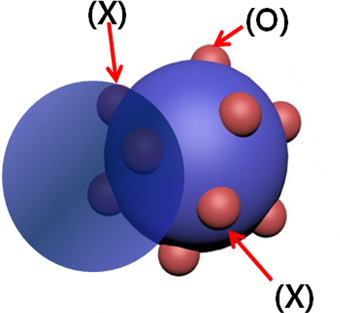


**Detailed calculation:** A full equation by Luo *et al*.S[[1]](#endnote-2) is the following:

$$\xi=\sqrt{\frac{-\frac{1}{\sqrt{2}}\mathrm{Re}\left[\frac{V_{u}r^{3}}{R^{3}}-\frac{2V_{u}r}{k^{2}R^{3}}(1-jkr)+\frac{V_{u}r}{k^{2}R^{3}}\exp\left(jk\left(R-r\right)\right)-j\frac{2V_{u}r}{kR^{2}}\exp\left(jk\left(R-r\right)\right)-\frac{2V_{u}r}{R}\exp\left(jk\left(R-r\right)\right)\right]}{V_{1}\frac{r^{3}}{R^{3}}}+1}$$

where ***ξ*** is the enhanced factor of the mass transfer coefficient (ratio of the mass transfer coefficients with and without ultrasonication) under a constant flow velocity ***V***_1_. ***V***_u_ is the amplitude of the vibration of the fluid flow. ***V*** = ***V***_1_ + ***V***_u_ cos(ωt). ***r*** is the particle radius. ***R*** is the particle plus boundary layer radius. ***k*** = 1/δ, with
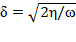
 as the thickness of the boundary layer in the ultrasonicated media.

We also derived a modified equation where experimental parameters are more visible:

where ***ξ*** is the enhanced factor of the mass transfer coefficient (ratio of the mass transfer coefficients with and without ultrasonication) under a constant flow velocity ***V***1. ***V***u is the amplitude of the vibration of the fluid flow. ***V*** = ***V***1 + ***V***u cos(ωt). ***r*** is the particle radius. ***R*** is the particle plus boundary layer radius. ***k*** = 1/δ, with
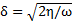
 as the thickness of the boundary layer in the ultrasonicated media.

1. **S1**. L. BenYi, L. YiGang, *Sci. China Ser. G*: *Physics, Mechanics & Astronomy,* 2008, **51,** 1496-1504. [↑](#endnote-ref-2)
